# Endoscopic Stenting and Clipping for Anastomotic Stricture and Persistent Tracheoesophageal Fistula after Surgical Repair of Esophageal Atresia in an Infant

**DOI:** 10.1155/2014/738981

**Published:** 2014-12-15

**Authors:** Mohammed Amine Benatta, Amine Benaired, Ahmed Khelifaoui

**Affiliations:** ^1^Digestive Endoscopy Unit, Military Central Hospital (HCA), 16000 Algiers, Algeria; ^2^Department of Paediatric Surgery, Military Central Hospital (HCA), 16000 Algiers, Algeria

## Abstract

Anastomotic stricture (AS) and recurrent tracheoesophageal fistula (TEF) are two complications of surgical repair of esophageal atresia (EA). Therapeutic endoscopic modalities include stenting, tissue glue, and clipping for TEF and endoscopic balloon dilation bougienage and stenting for esophageal strictures. We report herein a two-month infant with both EA and TEF who benefited from a surgical repair for EA, at the third day of life. Two months later he experienced deglutition disorders and recurrent chest infections. The esophagogram showed an AS and a TEF confirmed with blue methylene test at bronchoscopy. A partially covered self-expanding metal type biliary was endoscopically placed. Ten weeks later the stent was removed. This allows for easy passage of the endoscope in the gastric cavity but a persistent recurrent fistula was noted. Instillation of contrast demonstrated a fully dilated stricture but with a persistent TEF. Then we proceeded to placement of several endoclips at the fistula site. The esophagogram confirmed the TEF was obliterated. At 12 months of follow-up, he was asymptomatic. Stenting was effective to alleviate the stricture but failed to treat the TEF. At our knowledge this is the second case of successful use of endoclips placement to obliterate recurrent TEF after surgical repair of EA in children.

## 1. Introduction

Esophageal atresia (EA) remains the most common congenital anomaly of the esophagus and is associated with tracheoesophageal fistula (TEF) in the majority of cases. Following repair of EA early complications may occur including anastomotic stricture AS and recurrent TEF. Endoscopic modalities for the strictures include dilatation using either semirigid bougies (Savary-Gillard) or balloon dilatation (EDB) and stenting. The surgical treatment has always been an option in cases of intractable benign esophageal strictures. Recurrent TEF is a rare and difficult complication which can be repaired surgically using thoracoscopic or an open approach. TEF can also be repaired endoscopically by application of fibrin glue, stenting, and clipping. The Amplatzer devices have been used also in esophageal fistula. In children the stenting has been reported in both AS and TEF. We report a case of a two-month-old infant with both AS and recurrent TEF following surgical repair of EA treated with endoscopic stenting and clipping. To our knowledge this is the second case of successful closure of recurrent TEF with endoclips following surgical repair of EA in children.

## 2. Case Report

**Figure 1:**
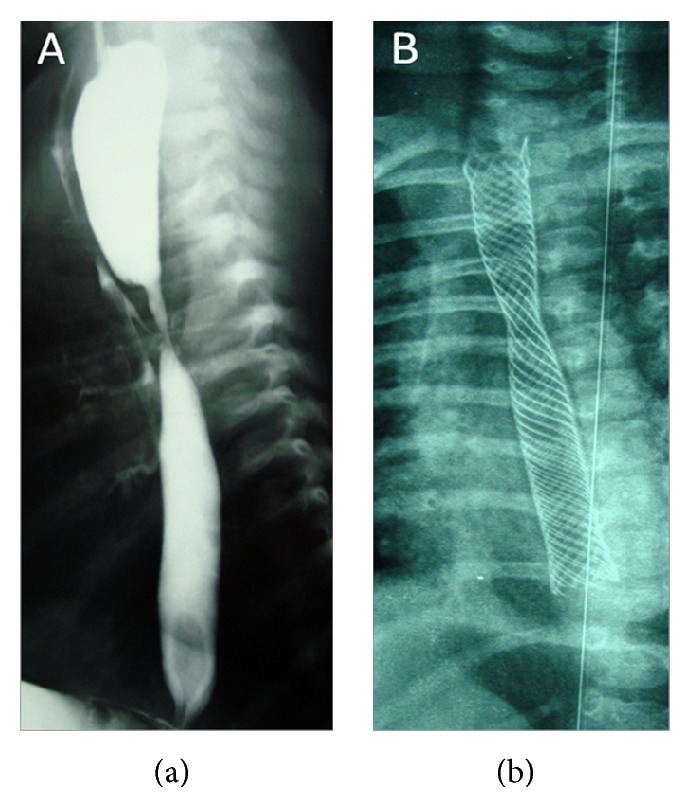
(a) The anastomotic stricture with the tracheoesophageal fistula. (b) The biliary SEMS in place covering the site of both anastomotic stricture and tracheoesophageal fistula.

An infant born with EA associated with distal TEF (type C) benefits at the third day of life from a surgical repair of his congenital developmental anomaly. At postoperative day 5 a recurrent TEF occurred and spontaneously closed 3 days later. Two months later our patient experienced cyanosis while feeding and recurrent chest infections with body weight loss. An AS with recanalization of the TEF was diagnosed at esophagogram (Figure 1(a)) and confirmed by bronchoscopy with blue methylene test. An endoscopic approach of these two complications with a self-expanding metal stent (SEMS) was performed. Under conscious sedation, following local pharyngeal anesthesia, the flexible endoscope was inserted into the esophagus until it reached the site of both the stricture and the recurrent fistula. Under combined endoscopic and fluoroscopic guidance, a partially covered self-expanding metal type biliary stent (Boston Scientific) 8 mm in diameter and 60 mm in length was placed in the mid to distal esophagus covering the area of the AS and the TEF as controlled on postoperative chest X-ray (Figure 1(b)).

**Figure 2:**
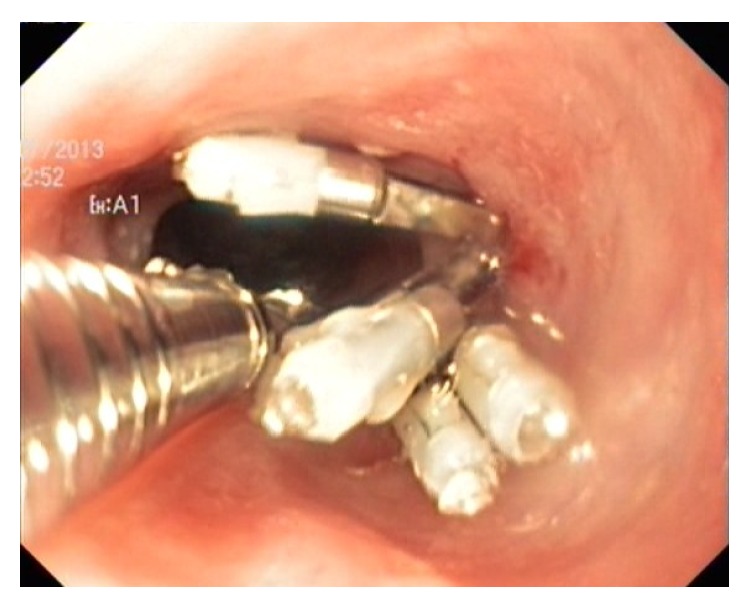
Endoclip placement at the persistent recurrent TEF site.

There were no procedure-related complications. Acid suppressive therapy was prescribed and refeeding started the next day. The stent was well tolerated and the patient was able to ingest liquids and soft food with improvement of respiratory signs. Four weeks later, chest X-ray was performed and demonstrated the stent was still in the proper position. The respiratory signs improved after placement of a feeding tube. Ten weeks after the stent placement this one was easily removed by using a foreign body snare. At endoscopy esophageal diameter appeared to be normal but with persistence of the recurrent fistula. Instillation of contrast, under fluoroscopy, demonstrated a fully dilated esophageal stricture with an evident TEF. Two weeks after stent removal, to close the persistent recurrent TEF, we proceeded to endoscopic placement of several endoclips (type easy clips; Olympus Optical Co., Ltd, Tokyo, Japan) at the fistula site. They are metal devices whose application is temporary “30 days” and the detachment is spontaneous (Figure 2). Esophagogram performed immediately after the procedure confirmed the TEF was obliterated. Our patient tolerates a full diet and was discharged. He was asymptomatic at 12 months of follow-up with normal growth and body weight.

## 3. Discussion

In our patient the stenting treatment was preferred, because we thought the risk of EBD including esophageal perforation to be especially high with the association of AS to TEF. Currently there is no definitive indication for placement of stents in children. The stents offer several advantages over traditional dilation techniques including the ability to provide continuous, radially oriented dilatation over a period of time. Most of the published literature regarding use of esophageal stents in children has involved patients with refractory caustic strictures or esophageal stricture after esophageal atresia repair [1]. The polyurethane covering of the stents prevents tissue ingrowth that facilitates removal and may also lead to fistula closure in our patient. In a premature child with both AS and blind mediastinal fistula at 10 days after surgical repair of EA, the placement of a covered metallic stent was effective for the two complications [2]. The available stents type which could be used in our small infant was the SEMS biliary one. Complications of stent placement include dysphagia, chest pain, gastroesophageal reflux, respiratory problems, esophageal perforation, stent migration, and hyperplastic tissue ingrowth or overgrowth which may occur at the ends of fully covered stents. Without any complication, stenting was effective to alleviate the AS even if it fails to treat the persistent recurrent TEF. Treatment of TEF with tissue glue is cheap but has a high failure rate and clipping can be difficult, especially when the fistula tract is mature. A combination of glue and clipping may improve the chances of successful closure of esophagomediastinal-trachea fistula after failure of fibrin glue although repeated attempts may be necessary [3]. In children the use of endoscopic clips for closure of esophageal fistula has been reported to be unsuccessful in one case [4] and successful in another more recent one [5]. In our patient endoclips placement was immediately effective to obliterate the persistent TEF. To our knowledge this is the second report of successful closure of recurrent TEF with endoclips following surgical repair of EA in children.

## 4. Conclusions

In our patient stenting was effective to alleviate the AS after surgical repair with only one biliary stent but failed to treat the TEF. Immediately in single stage endoclips placement was effective to obliterate the TEF. This endoscopic management allows for rapid discharge from the hospital and avoids the need for repeat thoracotomy or open surgery. This combination of two endoscopic modalities was safe and effective in achieving treatment of the two surgical complications of EA in this infant.
